# Comparing Additionality of Tuberculosis Cases Using GeneXpert or Smear-Based Active TB Case-Finding Strategies among Social Contacts of Index Cases in Nepal

**DOI:** 10.3390/tropicalmed8070369

**Published:** 2023-07-17

**Authors:** Suman Chandra Gurung, Kritika Dixit, Rajan Paudel, Manoj Kumar Sah, Ram Narayan Pandit, Tara Prasad Aryal, Shikha Upadhyay Khatiwada, Govind Majhi, Raghu Dhital, Puskar Raj Paudel, Gyanendra Shrestha, Bhola Rai, Gangaram Budhathoki, Mukti Khanal, Gokul Mishra, Jens Levy, Job Van de Rest, Anchal Thapa, Andrew Ramsay, Stephen Bertel Squire, Knut Lönnroth, Buddha Basnyat, Maxine Caws

**Affiliations:** 1 Birat Nepal Medical Trust, Kathmandu 44600, Nepal; suman.gurung@lstmed.ac.uk (S.C.G.); rajan@bnmt.org.np (R.P.); manoj@bnmt.org.np (M.K.S.); panditmph@gmail.com (R.N.P.); tarapsdaryal23@gmail.com (T.P.A.); shikha.khatiwada1992@gmail.com (S.U.K.); govind@bnmt.org.np (G.M.); raghu.dhital@bnmt.org.np (R.D.); puskar@bnmt.org.np (P.R.P.); gyanendra@bnmt.org.np (G.S.); bhola@bnmt.org.np (B.R.); gangaram@bnmt.org.np (G.B.); anchaltmagar@gmail.com (A.T.); maxine.caws@lstmed.ac.uk (M.C.); 2 Department of Clinical Sciences, Liverpool School of Tropical Medicine, Liverpool L35QA, UK; gokulmishra@gmail.com (G.M.); bertie.squire@lstmed.ac.uk (S.B.S.); 3 WHO Collaborating Centre on TB and Social Medicine, Department of Global Public Health, Karolinska Institutet, 10653 Stockholm, Sweden; knut.lonnroth@ki.se; 4 KNCV Tuberculosis Foundation, 2514 The Hague, The Netherlands; jenslevy@gmail.com (J.L.); job.vanrest@kncv.org.np (J.V.d.R.); 5 National TB Control Centre, Kathmandu 44600, Nepal; mukti6@gmail.com; 6 Division of Infection and Global Health, University of St Andrews, St Andrews KY169AJ, UK; andy.ramsay@st-andrews.ac.uk; 7 Oxford University Clinical Research Unit, Kathmandu 44600, Nepal; buddhabasnyat@gmail.com

**Keywords:** active case-finding, TB yield rate, ACF interventions, GeneXpert, Nepal

## Abstract

This study compares the yield and additionality of community-based active tuberculosis (TB) active case-finding strategies using either smear microscopy or GeneXpert as the TB diagnostic test. Active case-finding strategies screened social contacts of index cases and high-risk groups in four districts of Nepal in July 2017–2019. Two districts (Chitwan and Dhanusha) applied GeneXpert testing and two districts (Makwanpur and Mahotarri) used smear microscopy. Two control districts implemented standard national TB program activities. Districts implementing GeneXpert testing screened 23,657 people for TB, tested 17,114 and diagnosed 764 TB cases, producing a yield of 4.5%. Districts implementing smear microscopy screened 19,961 people for TB, tested 13,285 and diagnosed 437 cases, producing a yield of 3.3%. The screening numbers required were 31 for GeneXpert and 45.7 for smear districts. The test numbers required were 22.4 and 30.4 for GeneXpert and smear. Using the TB REACH additionality method, social contact tracing for TB through GeneXpert testing contributed to a 20% (3958/3322) increase in district-level TB notifications, smear microscopy 12.4% (3146/2798), and −0.5% (2553/2566) for control districts. Therefore, social contact tracing of TB index cases using GeneXpert testing should be implemented throughout Nepal within the TB FREE initiative to close the notification gap and accelerate progress toward END TB strategy targets.

## 1. Introduction

The World Health Organization (WHO) estimates that 10.6 million people developed tuberculosis (TB) disease in 2021, of which 4.2 million were either unreported or remained undiagnosed and untreated in 2021 [1]. TB was responsible for the deaths of 1.6 million people in 2021, the majority of which occurred among vulnerable people residing in low- and middle-income countries (LMICs) where health services are severely under resourced [1].

National TB programs (NTPs) in LMICs heavily rely on diagnosing TB through passive case-finding (PCF) and testing strategies for TB, principally using smear microscopy in government health facilities [2,3]. Delays to diagnosis caused by PCF are multifactorial and include barriers to accessing TB services, a lack of recognition of disease severity by the patient, the severe socioeconomic consequences of TB, and health system constraints in the LMICs [4,5]. In addition, smear microscopy has a sensitivity of only around 50% for active disease and, consequently, many patients receive a false-negative diagnosis, which leads to further delays in diagnosis and treatment [6]. Delays in prompt treatment initiation can lead to sustained transmission, poor health outcomes, resistance to TB drugs, or death [7,8]. Molecular testing approaches, such as GeneXpert, can simultaneously diagnose TB and detect resistance to rifampicin, a surrogate marker for muti-drug-resistant TB (MDR TB), which can significantly reduce time to enrolment for appropriate treatment for drug-resistant TB [9,10,11]. To accelerate the elimination of TB and achieve the END TB strategy target to reduce TB incidence by 90% from its 2015 baseline, a dramatically increased case notification is required [12,13]. WHO recommends both the scale-up of advanced molecular technology, such as GeneXpert, MTB/RIF, and the systematic screening of contacts of people with TB [14,15]. Evidence from LMICs has shown increased TB diagnosis using GeneXpert among household and close contacts in LMICs [15,16,17].

The systematic screening of contacts by the active case-finding (ACF) strategy is a global health priority and is identified as a key component of the END TB strategy for integrated, patient-centered care and prevention [12,13,18,19]. WHO defines ACF as ‘provider-initiated systematic screening and testing in the predetermined target groups, by assessing symptoms and using high accuracy tests, examinations and other procedures that can be applied rapidly’ [14]. ACF overcomes diagnostic barriers for the most vulnerable groups, reducing patient delay and pre-diagnosis costs for patients, and improving case-detection rates, case notification, and treatment outcomes [17,20]. Early diagnosis can shorten the infectious period, therefore reducing transmission, incidence, and prevalence [2,13,21,22,23,24]. However, without appropriate contextual strategies, ACF activities can be a financial burden for health systems and also contribute to stigma, discrimination, and increase false-positive and false-negative results [21,25,26]. ACF needs to be implemented with sustainable strategies based on the context, making optimal use of resources, balancing the potential benefits and challenges, and supplementing existing PCF strategies rather than replacing them [2,21,22,25,26,27].

Nepal is a low-income country with TB incidence of 245 per 100,000 population [28]. Although the NTP strategy 2016–2021 adopted both PCF and limited ACF approaches to diagnose new TB patients to achieve the END TB target for Nepal, a comprehensive strategy needs to be designed and implemented, which identifies the 40,000 people with TB who remain undiagnosed or unreported each year in Nepal [28,29]. While WHO stated that molecular diagnostics have higher sensitivity than smear microscopy, the costs, maintenance logistics, and training required are considerable challenges for the NTP, especially in remote rural Nepal. Therefore, despite the strong WHO recommendation based on global evidence that molecular-based TB diagnostic tools are used as the first diagnostic test for people being tested for TB, scale-up implementation in Nepal and similar resource-constrained health systems are slow. Evidence is required within the Nepali context of increased case finding using GeneXpert testing among social contacts of TB cases.

The IMPACT TB project was implemented in four districts of Nepal to determine if ACF using GeneXpert testing can increase TB case notifications at the district level. In this study, we compare the yield and additionality gained at the district level by applying two different implementation models of community-based active case-finding strategies in Nepal. Two districts (Makwanpur and Mahottari) applied smear microscopy for TB testing and two districts (Chitwan and Dhanusha) applied GeneXpert MTB/RIF among social contacts of TB cases and high-risk groups. The findings will inform intensification strategies for the scale-up results of ACF of TB in Nepal under the TB FREE Nepal initiative of the NTP.

## 2. Materials and Methods

### 2.1. Study Setting

From 1 November 2017 to 31 October 2019, the IMPACT TB project implemented ACF in four districts in Nepal: Chitwan (population 579,984; 2.2% of the national population), Makawanpur (population 420,477, 1.6% of national population), Mahottari (population 627,580, 2.4% of the national population), and Dhanusha (754,777, 2.8% of the national population) [30]. Dhanusha and Mahottari are flat-plain regions bordering India (known as the Terai in Nepal), while Makawanpur and Chitwan districts have a mixed terrain of hills and lowland Terai [31]. These districts were recommended by the NTP based on high-national-case notifications and the laboratory capacity to implement the study intervention.

**Figure 1:**
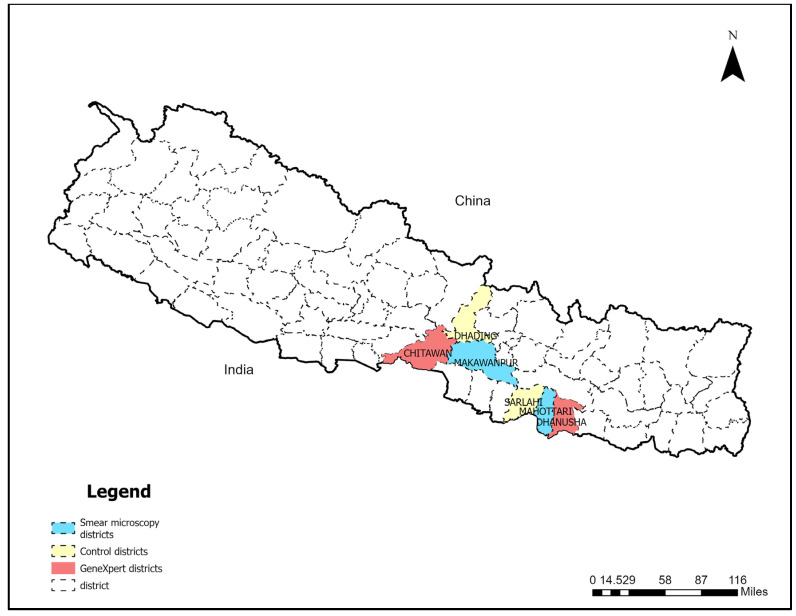
Map of Nepal showing intervention and control districts’ locations for IMPACT active TB case finding.

Dhading and Sarlahi were purposively selected as the two control districts implementing only routine National tuberculosis control program activities. Figure 1 shows the location of the study districts.

The study population included all social contacts of index TB patients who had been diagnosed in the 12 months preceding IMPACT TB implementation (January–December 2016), social contacts of newly identified index cases, and high-risk groups. During IMPACT TB implementation, the household contacts of index cases in the study districts were screened by the Global Fund supported activities of the NTP and were therefore not included in IMPACT TB case finding to avoid the duplication of TB services. Social contacts were individuals who did not belong to the same household, but had shared a close space with index TB patients during the three months prior to the diagnosis of the index case with TB [32]. We included adult contacts aged 18 years old and older who provided written informed consent for screening participation. Child contacts were referred to the appropriate NTP services for evaluation.

### 2.2. Intervention

All activities were implemented in consultation and coordination with the local health authorities. Thirty Community Health Volunteers (CHVs) were recruited in each project district and received training to identify and screen the social contacts of index cases with informed consent. A TB case-finding strategy was conducted in the four districts through three strategies: (a) close community contact tracing, (b) a small number of door-to-door screening in high-risk areas at the request of the district authorities, and (c) mobile TB diagnostic camps in hard-to-reach areas. In all cases, individuals were screened first by eight verbal questions to identify symptomatic individuals for testing. TB symptoms included in the questionnaire were the presence of a cough for more than 2 weeks, blood in cough, fever, night sweats, weight loss, chest pain, had TB in the past two years, and had contact with people with TB within six months.

Individuals responding positively to any one of the questions were advised and supported to take a test for TB. GeneXpert MTB/RIF was used for TB testing in Chitwan and Dhanusha districts. BNMT Nepal installed three GeneXpert machines in each of these districts at government health facilities strategically located to maximize population coverage for the district. GeneXpert locations were determined in consultation with the government health service personnel and community stakeholders at the district and provincial levels to ensure appropriate space and human resources to support the service. Makawanpur and Mahottari implemented ACF using standard NTP Zhiel–Neelsen smear microscopy for TB testing. BNMT Nepal conducted the servicing and maintenance of microscopes and supplied eleven new Olympus microscopes in these districts to replace faulty microscopes in the existing network. Training and refresher courses for laboratory staff in GeneXpert or Ziehl–Neelsen smear microscopy were conducted in the relevant districts.

#### 2.2.1. Close-Community Contact Tracing

A list of index patients, with both drug-susceptible and drug-resistant TB, was obtained from the government health facilities. These index cases were visited by CHVs at their homes and interviewed with consent to identify their social contacts for screening. Social contacts were then contacted, invited to participate, and if informed consent was granted, were verbally screened for TB symptoms using the eight-question symptom evaluation. Participants with one or more symptoms of TB were counseled to provide a sputum sample for testing, which was collected and transported to the nearest testing center by the CHV. In GeneXpert districts, one on the spot sample was collected and in microscopy districts two samples were collected; the first ‘on the spot’ and the second in the early morning of the following day. For each index case, whenever possible, at least ten social contacts were screened by the CHVs. The collected sputum samples were sent for free laboratory testing to either the microscopic center or GeneXpert center according to the intervention district. All those who tested positive were traced back and brought to the health facility for TB treatment free of cost under the NTP.

#### 2.2.2. TB Case-Finding Camps

Mobile TB case-finding camps were conducted targeting high-risk groups in geographically disadvantaged and remote communities with a high TB burden in the intervention districts. These camps were conducted in coordination with district public/health offices and local health facilities. CHVs conducted verbal community screening for TB symptoms. Sputum samples were collected and sent for testing either in GeneXpert or microscopic centers according to the intervention district. Those testing positive for TB were followed up by the respective CHVs and enrolled in the nearest Directly Observed Treatment Shortcourse (DOTS) center for treatment. These camps also included awareness counseling on TB signs, symptoms, and methods of prevention.

#### 2.2.3. Door-to-Door Screening in High-Risk Areas

At the request of the district authorities, a small number of door-to-door screening activities were conducted in high-TB risk areas, which supplemented the TB camp outreach activities. The CHVs conducted door-to-door visits in these areas and verbally screened for TB symptoms using the standard questionnaire. Symptomatic individuals were then tested by GeneXpert or smear according to the study district.

### 2.3. Ethical Approval

Ethical approval for the study was obtained from the Nepal Health Research Council 138/2017 and the Liverpool School of Tropical Medicine Research Ethics Committee (17-019). The study was approved by the Ministry of Health and Population and Social Welfare Council in Nepal and the District Health Office of each implementing district. Written informed consent was obtained from all the participants and all the identifying information was anonymized before analysis. Participants who did not provide consent for screening were encouraged to present to the routine TB diagnostic service at their nearest health facility.

### 2.4. Data Management and Analysis

The Koninklijke Nederlandse Centrale Vereniging tot bestrijding der Tuberculose (KNCV) TB Foundation and BNMT developed a custom web portal for data entry and management. Data from index case lists, screening forms, and laboratory forms were entered by the district-based community mobilizers trained by the KNCV TB Foundation. Data analysis was performed on R (version 3.5.1, R foundation for Statistical Computing).

#### TB Yield and Additionality Analysis

Notification rates were compared using official government notification data between districts implementing ACF using GeneXpert, ACF using smear microscopy testing, and districts implementing only the standard NTP activities.

Number needed to screen (NNS, total number of individuals verbally screened/number for individuals testing positive for TB) and number needed to test (NNT, number of individuals submitting a sputum sample for testing/number of individuals testing positive for TB) were calculated and compared for GeneXpert and smear microscopy ACF strategies.

TB cases are reported by the Nepal NTP using a trimester system (3 × four-month reporting periods each year), reporting each year from July 16–15 July.

We obtained official case notification data for the intervention and control districts from the NTP database.

To evaluate the additional TB case notification achieved overall by the project and disaggregated by the diagnostic method applied (smear microscopy or GeneXpert), we applied the additionality method used by TB REACH projects. This was first calculated by subtracting the cases notified during a baseline period two years previous to the intervention (16 July 2015–15 July 2017) from the cases notified during the period of active implementation (16 July 2017–15 July 2019) [33]. This was the crude additionality.

Second, to calculate the predicted case notification data for each district in the intervention years, we performed trend analysis using a linear regression method for the preceding three-year case notification data (16 July 2014–15 July 2017). We then applied the adjusted additionality method, which calculates the difference between the expected and recorded values during the months of implementation, to determine the adjusted additionality.

Finally, we created a generalized linear mixed-methods model fit to determine the relative increase associated with each intervention (GeneXpert or smear), while incorporating the variation arising from each district. The two-year baseline notification data were incorporated into the model. The model employed a Poisson’s distribution to model a TB notification over time and treated time as a fixed variable using the trimester variable.

## 3. Results

### Comparison of Yield from GeneXpert versus Smear Microscopy

In total, the IMPACT TB project verbally screened 43,618 for TB and diagnosed 1201 TB cases with microbiologically confirmed TB across all four districts. This was an overall project direct yield of 3.9% (*n* = 1201/30,399). The study also identified 28 rifampicin-resistant TB cases who were referred for further evaluation and treatment within the national TB program.

The two districts implementing GeneXpert testing for ACF (Dhanusha and Chitwan) screened a total of 23,657 people for TB, tested 17,114 and diagnosed 764 TB cases, producing a yield of 4.5%. The NNS in these districts was 31.0 and the NNT was 22.4.

The two districts implementing smear microscopy for ACF (Makwanpur and Mahotarri) screened a total of 19,961 people for TB, tested 13,285 and diagnosed 437 cases, producing an overall yield of 3.3%. The NNS in these districts was 45.7 and the NNT was 30.4.

Among the three ACF strategies implemented in the four districts, the majority of cases were identified through screening contacts of index cases: 33,767 people were screened from 3122 index cases through social contact tracing and tested 24,957 samples, which identified 1006 TB cases and produced an overall yield of 4%. From the 51 TB camps conducted and door-to-door screening, 5442 people were screened, which identified 195 TB cases and produce an overall yield of 3.6%. Twenty-seven camps and door-to-door screening using GeneXpert testing yielded 105 cases among 2460 tested (4.3% yield). Twenty-four camps and door-to-door screening using smear microscopy testing yielded 90 cases among 2982 tested (3.0% yield).

## 4. Additionality

Crude additional case notifications for each district and by testing strategy (GeneXpert vs. smear microscopy) are shown in Table 1. An overall additional crude notification of +22.6% was achieved for the GeneXpert districts, with a 0.5% decrease for the districts applying smear microscopy for ACF. During the same time period, the control districts reported a decline in the case notification of −3.6%.

**Figure 2:**
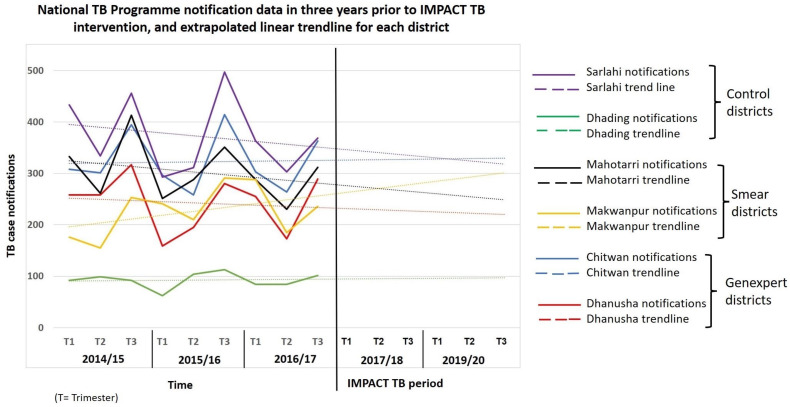
National TB program notification data by district in three years prior to IMPACT TB intervention and extrapolated linear trendline for each district.

**Figure 3:**
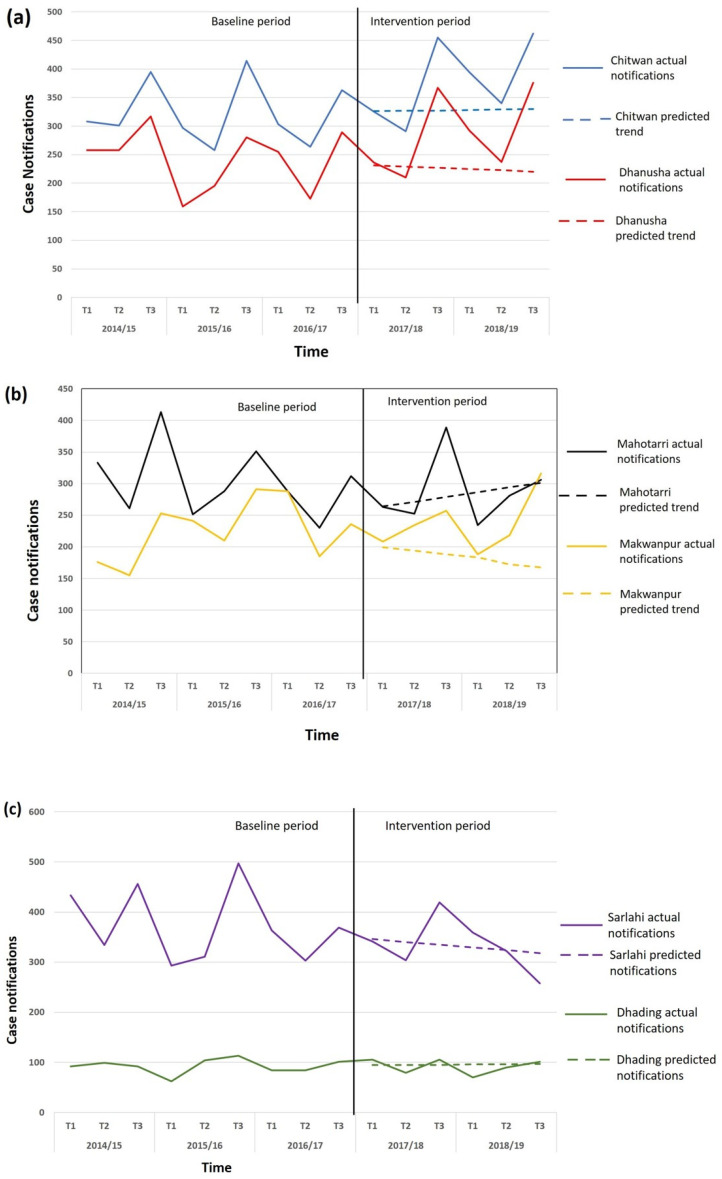
Achieved case notifications and predicted linear trend during the IMPACT TB intervention for (**a**) GeneXpert active case-finding districts, (**b**) smear microscopy active case-finding districts, and (**c**) control districts.

The predicted case notification for each district based on the trend for the three years preceding the IMPACT TB intervention is shown in Figure 2. Table 2 shows the predicted and actual notifications for each district, along with notifications aggregated by testing intervention (GeneXpert, smear or control), also shown graphically in Figure 3.

The dotted lines show the predicted linear trendline for years of IMPACT TB intervention. The analysis of the adjusted additionality of the case notifications using the trend analysis is calculated using the three-year baseline period.

Overall, the three-year trend extrapolation would predict a case notification total of 3322 for the GeneXpert districts, 2798 for the smear districts, and 2566 for the control districts. The actual notifications during the intervention period were 3985 for GeneXpert districts, 3146 for smear districts, and 2553 for the control districts. This produced an adjusted additionality of +20% (3985/3322) for the GeneXpert districts, +12.4% (3146/2798) for the smear districts, and −0.5% (2553/2566) for the control districts.

The adjusted additionality method (Table 2) revealed a strong variation between the two districts implementing smear microscopy, with Mahotarri district reporting a +56.4% increase in notifications compared to the predicted notification based on the trend. Makwanpur district, in contrast, reported a −16.2% decrease in actual notifications during the implementation period compared to the predicted trend.

The mixed-effects model estimated a relative increase associated with the implementation of a GeneXpert-based ACF of 1.15 [95% CI 1.07 to 1.25] and a relative increase associated with a smear-based ACF of 1.04 [95% CI 0.95 to 1.14]. Thus, there was a statistically significant increase of 15% in TB notifications attributable to the GeneXpert-based ACF, and a 4% increase in TB notifications in the smear-based ACF districts when fitting the data to the mixed effects model.

## 5. Discussion

Conducting active case-finding among social contacts and high-risk population groups using GeneXpert testing resulted in substantial additional yield and additionality at the district level in Nepal. An adjusted additionality of 20.0% was shown in the IMPACT TB districts applying GeneXpert testing, when using the TB REACH method to estimate adjusted additionality. Crude yield comparison (+22.6%) and the mixed-effects modeling (+15%) also showed a strong additionality for GeneXpert-based active case-finding outcomes. Strong additionality was shown for both the districts implementing GeneXpert testing, Chitwan and Dhanusha.

The crude yield comparison of notifications in both districts combined using smear microscopy testing showed a −0.5% decrease. However, when the adjusted additionality method was applied, which takes account of the three-year trend in the districts prior to the intervention, the smear districts showed an adjusted additionality of +12.4%. The mixed-effects modeling also showed a relatively small additionality for the smear districts of +4%. This was due to the decreasing trend in TB notifications in these districts prior to the IMPACT TB intervention. However, the two smear districts reported dramatically different results, with Mahotarri district showing an adjusted additionality of +56%, and Makwanpur showing a decrease of −16.2%. During the IMPACT TB intervention period, routine TB services in Makwanpur district were interrupted due to the sad death of the district TB and Leprosy Officer in a road traffic accident and a long delay in the appointment of a replacement. This variation in performance between the districts reflects the vulnerability of fragile health services to disruption and the need to build greater resilience into TB service delivery, particularly in rural areas [34]. The strong additionality in Mahottari district, by contrast, demonstrated that a substantial additional case notification could be achieved with smear microscopy testing within a robustly implemented program. A larger evaluation area including more districts in each arm of the study would have allowed a more robust analysis of the factors influencing the successful achievement of additionality.

This decreasing notification trend was also observed for the control districts (Sarlahi and Dhading) during the intervention period. Notifications in the control districts continued to decline by −3.6% during the two-year IMPACT TB intervention.

The number needed to screen and number needed to test were lower for GeneXpert testing compared to smear microscopy. For GeneXpert testing, 31 people were screened and 22 tested for every case detected, while for smear microscopy, 46 people were screened and 30 tested for every person diagnosed. This result was expected due to the known substantially higher sensitivity of GeneXpert testing for tuberculosis [15,16,35,36]. However, it is important to quantify the difference given the substantial difficulties of obtaining and transporting samples for TB testing in rural Nepal, and the higher costs of GeneXpert testing. The lower sensitivity of smear microscopy will generate a substantial number of false-negative results in people who have been screened for TB, thus wasting active case-finding resources, further delaying an accurate diagnosis and curative treatment and increasing the socioeconomic and psychosocial burden for people incorrectly diagnosed. A health system’s cost analysis of IMPACT TB interventions is being prepared for publication. GeneXpert testing is also able to identify multidrug-resistant TB (MDR TB) cases that smear microscopy is not [15]. Only one in five MDR TB cases in Nepal is presently diagnosed and treated through the national TB program [37]. The scale up of molecular tests such as GeneXpert MTB/RIF as the initial diagnostic test for people investigated for TB, as recommended by WHO, would close the detection gap for both drug susceptible and MDR TB cases.

The TB case notification gap in Nepal is approximately 40,000 cases per year, with only 40% of cases diagnosed and treated through the national TB program [37]. TB case detection plummeted due to the COVID-19 pandemic, a situation that was observed in many high-TB-burden countries [34,38,39,40,41]. Unfortunately, this resulted in TB deaths increasing globally for the first time in a decade, with 1.5 million deaths due to TB in 2020 [42]. Community-based patient-centered health services were shown to be more resilient than centralized services during the pandemic [34]. Active TB case-finding strategies through our network of community health workers reached the most vulnerable members of a community and supported them to access and complete treatment for TB. The active case-finding approach also reduces catastrophic health costs among households affected by TB [20,43,44]. The elimination of catastrophic health costs is one of three key targets of the STOP TB partnership’s END TB strategy, which Nepal has adopted [18,45].

For every dollar invested in TB control programs, there was an approximate USD 59 return on investment in LMICs [46]. This is one of the most cost-effective public health interventions. Delivering ‘people-centered care in the community’ and universally replacing smear microscopy with rapid molecular diagnostics as the initial diagnostic test are two priority actions of the STOP TB global plan to END TB 2020–2023 [46].

The IMPACT TB intervention analysis clearly shows that the ACF approaches applying GeneXpert testing result in substantial additional notifications at the district level. A recent systematic review of community-based ACF interventions found that results of ACF interventions are highly variable and achieving successful additionality depends on high-quality, robust, and high-intensity interventions [47]. ‘Scale-up of activities of ACF among high-risk and vulnerable groups’ is a priority of the five-year strategic plan for tuberculosis in Nepal, but no case notification target is defined among the key performance indicators [48]. ACF in Nepal should be intensified to include social contacts of index cases, as well as household contacts, and expanded to cover every district through GeneXpert testing to maximize case notification and close the diagnostic gap [19,24]. The National TB Control Centre has recently launched the TB FREE Nepal initiative in 53 municipalities, with a plan to scale this up to all 753 municipalities throughout the country within four years [45]. Sustained political commitment is necessary to achieve these ambitious goals. Comprehensive active case-finding strategies will be an essential component of increasing TB notification in Nepal and progressing towards the END TB goals. In addition, community-based active case-finding strategy supports equity of access to health services and health rights of people affected by TB through patient-centered comprehensive TB care [49].

### Limitations

The ACF intervention applied in this study was limited to bacteriologically confirmed TB. Due to the limited availability of radiologists in rural Nepal and budget constraints, we were not able to include referral pathways for clinical TB diagnoses for those with a negative smear or GeneXpert result. The inclusion of these strategies would certainly result in a higher yield of additional TB cases and should be further evaluated in Nepal.

The difference between the yield and additionality achieved between the microscopy districts (Mahottari and Makwanpur) highlighted the complexity of factors affecting successful ACF interventions. A larger study including cluster randomized district allocations would have provided more information on the factors underlying the successful achievement of additionality with smear microscopy-based ACF; however, it was unfortunately beyond the budget of this project.

## 6. Conclusions

Using the TB REACH additionality method, social contact tracing for TB through GeneXpert testing contributed to a 20% increase in district-level TB notifications in Nepal, while smear-microscopy-based ACF yielded 12.4% additionality. The social contact tracing of TB index cases using GeneXpert testing should be implemented throughout Nepal within the TB FREE initiative to close the notification gap and accelerate progress towards END TB strategy targets.

## Figures and Tables

**Table 1:** National TB program notification data for IMPACT TB intervention and control districts (crude data).

Category	District	Baseline						Intervention						Base-Line Total	Inter-Vention Total	% Change
		2015/16			2016/17			2017/18			2018/19					
		T1	T2	T3	T1	T2	T3	T1	T2	T3	T1	T2	T3			
**Evaluation—Xpert**																
	Chitwan	297	258	414	303	264	363	325	291	455	394	340	462	**1899**	**2267**	**+19.4**
	Dhanusha	159	195	280	255	173	289	236	210	367	292	237	376	**1351**	**1718**	**+27.2**
**Total**		456	453	694	558	437	652	561	501	822	686	577	838	**3250**	**3985**	**+22.6**
**Evaluation—Smear**																
	Mahottari	251	288	351	288	230	312	263	252	389	234	281	306	**1720**	**1725**	**+0.3**
	Makwanpur	241	201	291	288	185	236	208	234	257	188	218	316	**1442**	**1421**	**−1.5**
**Total**		492	489	642	576	415	548	471	486	646	422	499	622	**3162**	**3146**	**−0.5**
**Control**																
	Sarlahi	293	311	497	363	303	369	341	304	419	359	322	258	**2136**	**2003**	**−6.2**
	Dhading	62	104	113	84	84	101	105	79	105	70	90	101	**548**	**550**	**+0.4**
**Total**		355	415	610	447	387	470	446	383	524	429	412	559	**2648**	**2553**	**−3.6**

Footnote: this analysis of the crude additionality of the case notifications is calculated using a two-year baseline period.

**Table 2:** Predicted and actual case notifications by district and aggregated by testing strategy (smear or GeneXpert).

	Predicted						Actual Notified Cases in Intervention Period						Predicted Total	Inter-Vention Total	% Difference
District	2017/18		2018/19				2017/18			2018/19					
	T1	T2	T3	T1	T2	T3	T1	T2	T3	T1	T2	T3			
**Evaluation—GeneXpert**															
Chitwan	326	327	327	328	329	330	325	291	455	394	340	462	**1967**	**2267**	**+15.3**
Dhanusha	231	229	227	225	223	220	236	210	367	292	237	376	**1355**	**1718**	**+26.8**
**Total**	557	556	554	553	552	550	561	501	822	686	577	838	**3322**	**3985**	**+20.0**
**Evaluation—Smear**															
Mahotarri	199	194	188	183	172	167	263	252	389	234	281	306	**1103**	**1725**	**+56.4**
Makwanpur	264	271	279	286	294	301	208	234	257	188	218	316	**1695**	**1421**	**−16.2**
**Total**	463	465	467	469	466	468	471	486	646	422	499	622	**2798**	**3146**	**+12.4**
**Control**															
Sarlahi	346	340	335	329	324	318	341	304	419	359	322	258	**1992**	**2003**	**+0.5**
Dhading	95	95	95	96	96	97	105	79	105	70	90	101	**574**	**550**	**−4.2**
**Total**	441	435	430	425	420	415	446	383	524	429	412	559	**2566**	**2553**	**−0.5**

## Data Availability

The datasets used and/or analyzed during the present study are available from the corresponding author on reasonable request.

## Abbreviations

ACF	Active case-finding
BNMT	Birat Nepal Medical Trust
CHV	Community health volunteer
DOTS	Directly observed treatment shortcourse
KNCV	Koninklijke Nederlandse Centrale Vereniging tot bestrijding der Tuberculose
LMIC	Low- and middle-income countries
MDR	Multidrug resistant
NNS	Number needed to screen
NNT	Number needed to test
NTP	National tuberculosis program
PCF	Passive case-finding
TB	Tuberculosis
WHO	World Health Organization

## References

[1] World Health Organisation Global Tuberculosis Report 2021. https://www.who.int/publications/i/item/9789240037021.

[2] Ho J., Fox G.J., Marais B.J. (2016). Passive case finding for tuberculosis is not enough. Int. J. Mycobacteriol..

[3] Golub J.E., Mohan C.I., Comstock G.W., Chaisson R.E. (2005). Active case finding of tuberculosis: Historical perspective and future prospects. Int. J. Tuberc. Lung Dis..

[4] Waisbord S. (2006). Behavioural Barriers in Tuberculosis Control: A Literature Review. https://pdf.usaid.gov/pdf_docs/Pnadf406.pdf.

[5] de Vries S.G., Cremers A.L., Heuvelings C.C., Greve P.F., Visser B.J., Belard S., Janssen S., Spijker R., Shaw B., Hill R.A. (2017). Barriers and facilitators to the uptake of tuberculosis diagnostic and treatment services by hard-to-reach populations in countries of low and medium tuberculosis incidence: A systematic review of qualitative literature. Lancet Infect. Dis..

[6] Steingart K.R., Ramsay A., Pai M. (2007). Optimizing sputum smear microscopy for the diagnosis of pulmonary tuberculosis. Expert Rev. Anti-Infect. Ther..

[7] Shewade H.D., Gupta V., Satyanarayana S., Pandey P., Bajpai U.N., Tripathy J.P., Kathirvel S., Pandurangan S., Mohanty S., Ghule V.H. (2019). Patient characteristics, health seeking and delays among new sputum smear positive TB patients identified through active case finding when compared to passive case finding in India. PLoS ONE.

[8] Vo L.N.Q., Forse R.J., Codlin A.J., Vu T.N., Le G.T., Do G.C., Van Truong V., Dang H.M., Nguyen L.H., Nguyen H.B. (2020). A comparative impact evaluation of two human resource models for community-based active tuberculosis case finding in Ho Chi Minh City, Viet Nam. BMC Public Health.

[9] MacLean E., Kohli M., Weber S.F., Suresh A., Schumacher S.G., Denkinger C.M., Pai M. (2020). Advances in Molecular Diagnosis of Tuberculosis. J. Clin. Microbiol..

[10] Cavanaugh J.S., Modi S., Musau S., McCarthy K., Alexander H., Burmen B., Heilig C.M., Shiraishi R.W., Cain K. (2016). Comparative Yield of Different Diagnostic Tests for Tuberculosis among People Living with HIV in Western Kenya. PLoS ONE.

[11] Zenteno-Cuevas R. (2022). New molecular mechanisms related to drug resistance in tuberculosis. Microbes Infect. Chemother..

[12] World Health Organisation Global Strategy and Targets for Tiberculosis Prevention, Care and Control after 2015: Report by the Sceretariat. https://apps.who.int/iris/handle/10665/172828.

[13] Houben R., Menzies N.A., Sumner T., Huynh G.H., Arinaminpathy N., Goldhaber-Fiebert J.D., Lin H.H., Wu C.Y., Mandal S., Pandey S. (2016). Feasibility of achieving the 2025 WHO global tuberculosis targets in South Africa, China, and India: A combined analysis of 11 mathematical models. Lancet Glob. Health.

[14] World Health Organisation Consolidated Guidelines on Tuberculosis Module 2: Screening—Systematic Screening for Tuberculosis Disease. https://www.who.int/publications/i/item/9789240022676.

[15] World Health Organisation Consolidated Guidelines on Tuberculosis. Module 3: Diagnosis—Rapid Diagnostics for Tuberculosis Detection 2021 Update. https://www.who.int/publications/i/item/9789240029415.

[16] World Health Organization (2013). Systematic Screening for Active Tuberculosis: Principles and Recommendations. https://www.who.int/tb/publications/Final_TB_Screening_guidelines.pdf.

[17] Gurung S.C., Dixit K., Rai B., Dhital R., Paudel P.R., Acharya S., Budhathoki G., Malla D., Levy J.W., Lonnroth K. (2021). Comparative Yield of Tuberculosis during Active Case Finding Using GeneXpert or Smear Microscopy for Diagnostic Testing in Nepal: A Cross-Sectional Study. Trop. Med. Infect. Dis..

[18] World Helath Organisation Gear up to END TB: Introducing the END TB Stratgey. https://apps.who.int/iris/handle/10665/156394.

[19] Bohlbro A.S., Hvingelby V.S., Rudolf F., Wejse C., Patsche C.B. (2021). Active case-finding of tuberculosis in general populations and at-risk groups: A systematic review and meta-analysis. Eur. Respir. J..

[20] Gurung S.C., Rai B., Dixit K., Worrall E., Paudel P.R., Dhital R., Sah M.K., Pandit R.N., Aryal T.P., Majhi G. (2021). How to reduce household costs for people with tuberculosis: A longitudinal costing survey in Nepal. Health Policy Plan..

[21] Biermann O., Atkins S., Lonnroth K., Caws M., Viney K. (2020). ‘Power plays plus push’: Experts’ insights into the development and implementation of active tuberculosis case-finding policies globally, a qualitative study. BMJ Open.

[22] Biermann O., Kluppelberg R., Lonnroth K., Viney K., Caws M., Atkins S. (2021). ‘A double-edged sword’: Perceived benefits and harms of active case-finding for people with presumptive tuberculosis and communities-A qualitative study based on expert interviews. PloS ONE.

[23] Yuen C.M., Amanullah F., Dharmadhikari A., Nardell E.A., Seddon J.A., Vasilyeva I., Zhao Y., Keshavjee S., Becerra M.C. (2015). Turning off the tap: Stopping tuberculosis transmission through active case-finding and prompt effective treatment. Lancet.

[24] Stop TB Partnership (2019). The Global Plan to Stop TB: 2018–2022. The Paradigm Shift. https://stoptb.org/assets/documents/global/plan/GPR_2018-2022_Digital.pdf.

[25] Saunders M.J., Tovar M.A., Collier D., Baldwin M.R., Montoya R., Valencia T.R., Gilman R.H., Evans C.A. (2019). Active and passive case-finding in tuberculosis-affected households in Peru: A 10-year prospective cohort study. Lancet Infect. Dis..

[26] Lin H.H., Dowdy D., Dye C., Murray M., Cohen T. (2012). The impact of new tuberculosis diagnostics on transmission: Why context matters. Bull. World Health Organ..

[27] Biermann O., Tran P.B., Forse R.J., Vo L.N.Q., Codlin A.J., Viney K., Caws M., Lonnroth K. (2021). Capitalizing on facilitators and addressing barriers when implementing active tuberculosis case-finding in six districts of Ho Chi Minh City, Vietnam: A qualitative study with key stakeholders. Implement. Sci..

[28] Ministry of Health and Population (2020). National TB Prevalence Survey 2018/19. https://nepalntp.gov.np/wp-content/uploads/2021/03/NTPS-Report-Bodypages.pdf.

[29] National Tuberculosis Center (2016). National Strategic Plan for Tuberculosis Prevention, Care and Control. Kathmandu. https://nepalntp.gov.np/wp-content/uploads/2018/01/NSP-report-english-revised.pdf.

[30] Central Bureau of Statistics (2012). National Population and Housing Census 2011 (National Report).

[31] National Tuberculosis Program Nepal (2020). Annual Report 2075/76 (2018/19).

[32] World Health Organization (2021). WHO Operational Handbook on Tuberculosis. Module 2: Screening—Systematic Screening for Tuberculosis Disease [M/OL].

[33] Creswell J., Sahu S., Blok L., Bakker M.I., Stevens R., Ditiu L. (2014). A multi-site evaluation of innovative approaches to increase tuberculosis case notification: Summary results. PLoS ONE.

[34] Arsenault C., Gage A., Kim M.K., Kapoor N.R., Akweongo P., Amponsah F., Aryal A., Asai D., Awoonor-Williams J.K., Ayele W. (2022). COVID-19 and resilience of healthcare systems in ten countries. Nat. Med..

[35] Shapiro A.E., Ross J.M., Yao M., Schiller I., Kohli M., Dendukuri N., Steingart K.R., Horne D.J. (2021). Xpert MTB/RIF and Xpert Ultra assays for screening for pulmonary tuberculosis and rifampicin resistance in adults, irrespective of signs or symptoms. Cochrane Database Syst. Rev..

[36] Zifodya J.S., Kreniske J.S., Schiller I., Kohli M., Dendukuri N., Schumacher S.G., Ochodo E.A., Haraka F., Zwerling A.A., Pai M. (2021). Xpert Ultra versus Xpert MTB/RIF for pulmonary tuberculosis and rifampicin resistance in adults with presumptive pulmonary tuberculosis. Cochrane Database Syst. Rev..

[37] World Health Organisation Tuberculosis Profile: Nepal. https://worldhealthorg.shinyapps.io/tb_profiles/?_inputs_&entity_type=%22country%22&lan=%22EN%22&iso2=%22NP%22.

[38] Pai M., Kasaeva T., Swaminathan S. (2022). COVID-19’s Devastating Effect on Tuberculosis Care—A Path to Recovery. N. Engl. J. Med..

[39] Dheda K., Perumal T., Moultrie H., Perumal R., Esmail A., Scott A.J., Udwadia Z., Chang K.C., Peter J., Pooran A. (2022). The intersecting pandemics of tuberculosis and COVID-19: Population-level and patient-level impact, clinical presentation, and corrective interventions. Lancet Respir. Med..

[40] Ruhwald M., Carmona S., Pai M. (2021). Learning from COVID-19 to reimagine tuberculosis diagnosis. Lancet Microbe.

[41] Zimmer A.J., Klinton J.S., Oga-Omenka C., Heitkamp P., Nawina Nyirenda C., Furin J., Pai M. (2022). Tuberculosis in times of COVID-19. J. Epidemiol. Community Health.

[42] Kirby T. (2021). Global tuberculosis progress reversed by COVID-19 pandemic. Lancet Respir. Med..

[43] Dixit K., Biermann O., Rai B., Aryal T.P., Mishra G., Teixeira de Siqueira-Filha N., Paudel P.R., Pandit R.N., Sah M.K., Majhi G. (2021). Barriers and facilitators to accessing tuberculosis care in Nepal: A qualitative study to inform the design of a socioeconomic support intervention. BMJ Open.

[44] Gurung S.C., Dixit K., Rai B., Caws M., Paudel P.R., Dhital R., Acharya S., Budhathoki G., Malla D., Levy J.W. (2019). The role of active case finding in reducing patient incurred catastrophic costs for tuberculosis in Nepal. Infect. Dis. Poverty.

[45] Reinvigorating Efforts to END TB in Nepal. https://www.who.int/nepal/news/detail/09-02-2022-Reinvigorating-efforts-to-end-Tuberculosis-in-Nepal.

[46] STOP TB Partnership Global Plan to End TB 2023–2030. https://omnibook.com/embedview/dc664b3a-14b4-4cc0-8042-ea8f27e902a6/en?no-ui.

[47] Burke R.M., Nliwasa M., Feasey H.R., Chaisson L.H., Golub J.E., Naufal F., Shapiro A.E., Ruperez M., Telisinghe L., Ayles H. (2021). Community-based active case-finding interventions for tuberculosis: A systematic review. Lancet Public Health.

[48] (2022). National Strategic Plan to End Tuberculosis (2021/22–2025/26).

[49] STOP TB Partnership Declaration of the Rights of People Affected by TB. May 2019. https://stoptb.org/assets/documents/communities/FINAL%20Declaration%20on%20the%20Right%20of%20People%20Affected%20by%20TB%2013.05.2019.pdf.

